# Nosological problems in psychiatric diagnosis – Factitious disorder, Conversion, and Simulation

**DOI:** 10.1192/j.eurpsy.2025.235

**Published:** 2025-08-26

**Authors:** F. Novais, D. Telles-Correia

**Affiliations:** Faculdade de Medicina, Lisboa, Portugal

## Abstract

**Abstract:**

Diagnosing psychiatric conditions that involve the intentional or unconscious production of symptoms remains a significant challenge in clinical practice. This presentation examines a clinical case that highlights the difficulties in distinguishing between Factitious Disorder, Conversion Disorder, and Simulation. A 34-year-old woman was admitted with sudden onset of neurological symptoms, including pseudo epileptic crises. Her symptoms fluctuated inconsistently with clinical observation and failed to correlate with established neurological patterns, raising the suspicion of Conversion Disorder. However, further investigation revealed inconsistencies in her medical history and a pattern of seeking unnecessary treatments, suggesting the possibility of Factitious Disorder. Additionally, external incentives, such as the potential for financial compensation, prompted consideration of Simulation. The case presents a diagnostic dilemma that underscores the overlapping features of these conditions.

Neuroimaging may provide valuable insights into the case, supporting the exclusion of neurological pathologies but also subtle changes in brain activity in the areas involved in emotional regulation and self-representation, which may suggest the involvement of underlying psychological factors common in both Conversion and Factitious Disorder.

This case exemplifies the critical nosological challenges in differentiating between Factitious Disorder, Conversion Disorder, and Simulation. It highlights the importance of a comprehensive clinical approach, including neuroimaging, thorough psychological assessment, and consideration of psychosocial factors. The discussion aims to deepen understanding of these complex disorders and promote more accurate and nuanced diagnostic practices in psychiatry.

**Disclosure of Interest:**

None Declared

